# Palmatine-Intercalated Montmorillonite Improves Intestinal Health and Attenuates Systemic Inflammation in ETEC-Challenged Piglets

**DOI:** 10.3390/ani16182868

**Published:** 2026-09-11

**Authors:** Liping Gan, Yifeng Zhao, Shiya Li, Yingying Sheng, Dongtian Zhao, Yuan Yuan, Hanzhen Qiao, Peng Wang, Erzhen Duan, Tengpeng Liu, Jinrong Wang

**Affiliations:** School of Bioengineering, Henan University of Technology, Zhengzhou 450001, China; lpgan@haut.edu.cn (L.G.);

**Keywords:** ETEC, piglets, palmatine–montmorillonite composite, antibacterial, anti-inflammatory

## Abstract

Enterotoxigenic *Escherichia coli* (ETEC) infection severely impairs post-weaning piglet health, causing intestinal dysfunction, systemic inflammation, and significant economic losses in the swine industry. This study aims to evaluate a synthesized palmatine-intercalated montmorillonite composite (MP) for improving growth performance, immune response, and intestinal health in ETEC-challenged piglets. Following structural characterization, an in vivo trial was conducted using 30 crossbred piglets (Yorkshire × Landrace × Duroc; 15.12 ± 2.95 kg BW) challenged with ETEC K88 and supplemented with 0.02%, 0.5%, and 1.5% MP. Structural analysis confirmed successful intercalation of palmatine into montmorillonite, with enhanced antibacterial and anti-inflammatory properties. In vivo, dietary inclusion of the 1.5% MP significantly alleviated ETEC-induced growth depression, as evidenced by increased average daily gain and improved apparent total tract digestibility of crude protein. These effects were associated with improved jejunal morphology and upregulation of *PEPT1*, *B0AT1*, and *SGLT1*. Moreover, MP supplementation attenuated systemic inflammation and hepatic stress, as indicated by reduced serum AST, TNF-α, and IL-1β levels. Microbiota analysis showed that MP modulated ETEC-associated alterations in the cecal microbial community. In conclusion, the MP represents a promising nutritional approach to improve intestinal resilience, nutrient utilization, and growth performance of ETEC-challenged piglets.

## Simple Summary

This study developed a new feed additive by combining montmorillonite and palmatine to improve their beneficial properties. The composite helped protect piglets from infection-related intestinal damage, reduced inflammation, and improved nutrient utilization. These findings indicate that the montmorillonite–palmatine composite has potential as a nutritional strategy to support piglet health, improve production efficiency, and reduce reliance on conventional antimicrobial approaches.

## 1. Introduction

The post-weaning period is a critical stage in pig production, characterized by abrupt changes in diet, environment, and social structure that predispose piglets to intestinal dysfunction and enteric disease [1,2,3]. Enterotoxigenic *Escherichia coli* (ETEC) is the most prevalent pathogen associated with post-weaning diarrhea. It damages intestinal barrier function and triggers an inflammatory response, which severely impacts animal welfare and production performance, resulting in significant economic losses in the swine industry [4,5]. ETEC colonizes the small intestine via fimbrial adhesion, secretes enterotoxins that disrupt electrolyte balance, and induces villus atrophy and inflammation, ultimately impairing barrier function and nutrient absorption [6,7,8]. These pathological changes are accompanied by increased systemic inflammatory markers, including IL-6, TNF-α, and IL-8, leading to reduced growth performance and economic losses [9,10]. The gut microbiota plays a central role in maintaining intestinal function, nutrient utilization, and immune homeostasis [11]. ETEC infection disrupts microbial composition and function, contributing to inflammation and impaired digestion [7,12]. This dysbiosis can further promote pathogen persistence and exacerbate intestinal damage, highlighting the need for nutritional strategies that stabilize the gut ecosystem of the piglets.

Antibiotic growth promoters (AGPs) were historically used to control post-weaning diarrhea and support growth performance [13]. However, increasing concerns regarding antimicrobial resistance and residues have led to their restriction or ban in many regions, including the European Union and China. This regulatory shift has created an urgent demand for sustainable nutritional alternatives that support gut health and confer resilience against pathogenic challenges [14].

Montmorillonite (MMT), a layered silicate mineral, is widely used as a feed additive due to its adsorption and ion-exchange properties [15,16]. MMT selectively adsorbs bacteria and toxins, preventing their adherence to the intestinal wall and protecting the host’s barrier function. Supplementation at low levels (0.2% to 1.0%) has been shown to improve intestinal morphology, nutrient digestibility, and microbial composition [17]. However, its non-specific adsorption may increase the risk of binding essential micro-nutrients in the diet of piglets, thereby compromising nutritional efficacy. Furthermore, high doses of MMT (typically exceeding 2%) have been shown to induce oxidative stress and decrease mineral deposition in pigs [18]. Additionally, unmodified MMT exhibits limited intrinsic anti-inflammatory properties. These limitations have prompted the development of modified MMT-based composites to enhance functional specificity and efficacy [15,19,20].

Palmatine (PAL), a naturally occurring isoquinoline alkaloid, possesses potent biological activities, including robust anti-inflammatory, antioxidant, and antibacterial effects [21]. PAL has been shown to alleviate diarrhea and weight loss in infection models by exerting anti-inflammatory effects and maintaining intestinal barrier integrity [22]. Moreover, PAL can reshape the intestinal microenvironment and alleviate gut dysbiosis by restoring microbial homeostasis [17,22]. However, the clinical utility of PAL is heavily restricted by its low oral bioavailability due to poor intestinal absorption and rapid efflux mediated by P-glycoprotein [23].

While both MMT and PAL show promise individually, combining MMT with PAL may integrate adsorption capacity with targeted bioactivity while improving PAL delivery. However, the effects of such a composite on intestinal function and microbial stability under ETEC challenge remain unclear. Therefore, this study aimed to synthesize a palmatine-intercalated montmorillonite composite (MP) and evaluate its effects on growth performance, intestinal morphology, and gut microbiota in ETEC K88-challenged weaned piglets, thereby providing a basis for its application as a functional alternative in swine health management.

## 2. Materials and Methods

### 2.1. Synthesis of the MP

**Figure 1:**
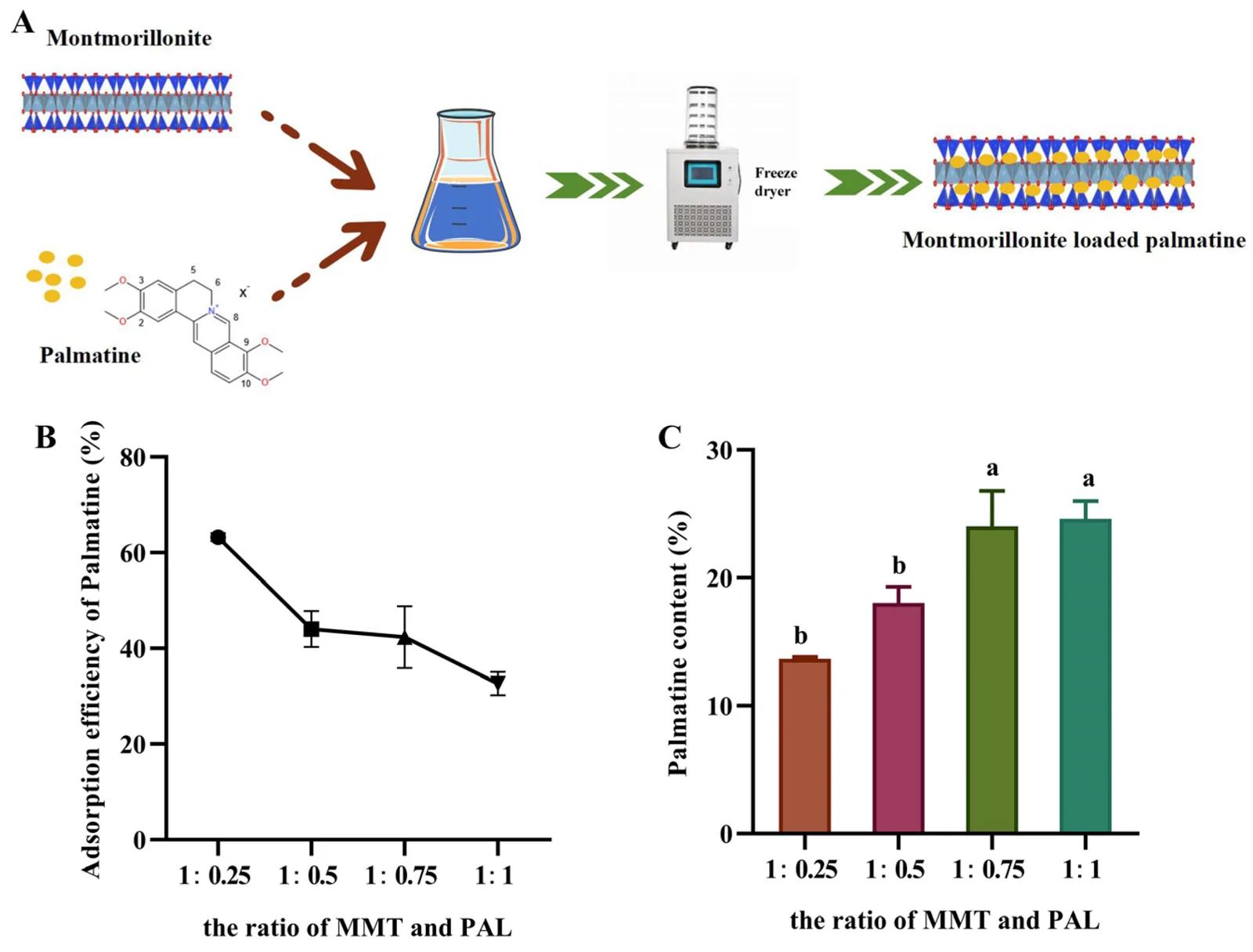
Synthesis of the palmatine-montmorillonite composite (MP). (**A**) Schematic illustration of the MP synthesis process; (**B**) adsorption efficiency of palmatine at various montmorillonite-to-palmatine (MMT:PAL) ratios; and (**C**) palmatine content within the MP at different MMT:PAL ratios. Different lowercase letters (a, b) indicate significant differences (*p* < 0.05). MMT, montmorillonite; PAL, palmatine.

Prior to the synthesis of the MP, raw MMT (Tianyu Technology Company, Chifeng, China) underwent a purification process adapted from the protocols described by Qiao et al. [24]. A wet dispersion method was employed to facilitate the efficient intercalation of PAL (purity ≥ 97%, Macklin, Shanghai, China) into the MMT layers. MP were synthesized at PAL-to-MMT mass ratios of 0.25:1, 0.5:1, 0.75:1, and 1:1. The supernatant was analyzed via HPLC to determine the concentration of unbound PAL and to calculate the adsorption efficiency. Finally, the collected pellet was washed twice with deionized water, re-centrifuged to eliminate non-specifically adsorbed PAL, and lyophilized in a vacuum freeze-dryer (Figure 1A).

### 2.2. Structural Characterization of the MP

The microstructure and morphology of MMT, PAL, and the MP were examined using a scanning electron microscope (SEM; SU8200, Hitachi, Tokyo, Japan). Approximately 0.1 g of powder was affixed to an SEM stub and sputter-coated with gold prior to imaging, which was conducted at an accelerating voltage of 3 kV at magnifications of 1000× and 10,000×. X-ray diffraction (XRD) patterns were acquired using a diffractometer (Empyrean, PANalytical, Almelo, The Netherlands) equipped with Cu Kα radiation (λ = 1.5406 Å) operating at 40 kV and 30 mA. Lyophilized samples were smear-mounted onto glass slides, and data were collected from 5° to 60° (2θ) at a scan rate of 5°/min. Interlayer d-spacing values were calculated from the XRD peak positions using Bragg’s law [25]. Fourier-transform infrared (FTIR) spectroscopy was performed on MMT, PAL, and the MP using a Nicolet iS20 spectrometer (Thermo Scientific, Waltham, MA, USA).

### 2.3. Quantification of Palmatine Release Kinetics

To evaluate the release properties of the MP under simulated porcine gastrointestinal conditions, simulated gastric and intestinal fluids were prepared. The simulated gastric fluid was prepared by mixing 0.2 M KCl and 0.2 M HCl and adjusting the solution to pH 4.0. The simulated intestinal fluid was prepared by mixing 0.1 M NaOH and 0.1 M KH_2_PO_4_ and adjusting the solution to pH 7.4. The release kinetics of PAL from the MP under these simulated gastric and intestinal conditions were determined using dialysis bags (molecular weight cut-off, 8000–14,000 Da). Briefly, 10 mg of MP was suspended in 10 mL of either a simulated gastric or enteric buffer within the dialysis bag, which was subsequently submerged in 90 mL of the corresponding dissolution medium. The system was maintained at 37 °C with constant agitation. To determine the cumulative release rate, 0.3 mL of the external dissolution medium was collected at specific intervals and immediately replaced with an equal volume of fresh buffer to maintain sink conditions.

### 2.4. Antibacterial Activity of the MP

To evaluate the inhibitory effects of the MP against pathogenic bacteria, *Escherichia coli* ETEC (CICC 10667) and *Staphylococcus aureus* (CICC AB 91093) were utilized. The concentrations of MMT, PAL, and the MP were set at 4 mg/mL for the *E. coli* assays and 0.2 mg/mL for the *S. aureus* assays. Briefly, 100 μL of bacterial suspension (10^8^ CFU/mL) was added to 4.9 mL of LB broth containing MMT, PAL, or the MP; the control group consisted of LB broth without additives. The mixtures were incubated at 37 °C with shaking for 2 h. Subsequently, 100 μL of each solution was collected, subjected to appropriate serial dilution, and plated onto agar for bacterial colony counting. Six independently inoculated bacterial cultures were analyzed for each treatment (*n* = 6).

### 2.5. Effects of MP on LPS-Induced Inflammation in IPEC-J2 Cells

Based on the cytotoxicity results, appropriate non-toxic concentrations were selected to investigate the anti-inflammatory effects of the MP against LPS-induced injury. IPEC-J2 cells were seeded into 24-well plates and pretreated with 100 μg/mL of PAL, MMT, or MP for 24 h, followed by exposure to 10 μg/mL of LPS for 12 h. The culture supernatants were collected, and the concentrations of the pro-inflammatory cytokines tumor necrosis factor-alpha (TNF-α) and interleukin-1 beta (IL-1β) were quantified using ELISA kits (TNF-α, CSB-E16980p; IL-1β, CSB-E06782p; Cusabio, Wuhan, China) according to the manufacturer’s instructions. Three independent biological replicates were performed for each treatment (*n* = 3 wells per treatment).

### 2.6. Animals, Diets, and Experimental Design

To evaluate the efficacy of the MP in alleviating ETEC-induced diarrhea and inflammation in vivo, 30 healthy crossbred piglets (Yorkshire × Landrace × Duroc) with an initial average body weight (BW) of 15.12 ± 2.95 kg (49 ± 1 d of age) were used. The piglets were obtained from a commercial pig farm, weaned at 30 d of age, and transferred to the experimental facility at approximately 35 d of age, followed by a 14 d adaptation period before the experiment commenced. The piglets were clinically healthy at enrollment, with equal numbers of male and female piglets evenly distributed among the 5 treatment groups. Thirty piglets were randomly assigned to 5 treatment groups, with 6 piglets per group. The sample size was selected based on the number of animals used in previous ETEC challenge studies in weaned piglets, the expected biological variability of post-weaning animals, and practical considerations related to animal housing and experimental capacity. Throughout the 21 d study, the environmental temperature was maintained at 25 °C and the relative humidity at 55%. Each piglet was housed individually in a pen (1.30 m × 0.5 m) equipped with a single-side feeder and a nipple drinker to provide ad libitum access to feed and water. The basal diets were formulated to meet or exceed NRC (2012) [26] nutritional requirements for nutrients and energy (Table 1). The control group was fed the basal diet alone, while the four remaining groups were challenged with ETEC K88 and fed the basal diet supplemented with 0, 0.02%, 0.5%, or 1.5% MP. On days 19, 20, and 21, feces were collected from each pen, and feed consumed was recorded to determine nutrient digestibility. On days 17, 18, and 20, piglets in the ETEC-challenged groups received 30 mL of an ETEC K88 suspension (5 × 10^10^ CFU/mL, O149:K91, K88-positive, LT/STa/STb-producing, obtained from China General Microbiological Culture Collection Center) via oral gavage using a syringe attached to a soft tube. Piglets in the control group received an equivalent volume of sterile saline. To minimize the risk of cross-contamination between ETEC-challenged and non-challenged piglets, all piglets were housed individually in separate pens, with the experimental groups physically separated. Separate personnel and dedicated equipment were used for the challenged and non-challenged groups. Appropriate protective materials, including disposable gloves and protective clothing, were used and changed between groups. Pens and equipment were routinely cleaned and disinfected throughout the experimental period. Piglet body weight and feed disappearance were recorded on a pen basis on days 0, 7, 14, and 21 to calculate the average daily gain (ADG), average daily feed intake (ADFI), and feed to gain (F:G) ratio.

### 2.7. Sample Collection

On day 21, blood samples were collected from the jugular vein of all piglets (*n* = 6 per treatment group). The blood samples were allowed to clot at room temperature for 2 h, followed by centrifugation at 3000× *g*, 4 °C for 15 min to obtain serum. The resulting serum was aliquoted into pre-sterilized, low-binding vials and stored at −80 °C for further analysis. Following blood collection, all piglets were euthanized to facilitate tissue sampling. For histological analysis, 0.5 cm segments from the same relative locations of the jejunum were excised, rinsed with phosphate-buffered saline (PBS), and fixed in 4% paraformaldehyde. Simultaneously, additional segments of the jejunum were collected and divided into three pieces; the jejunal mucosa was gently scraped using a sterile glass slide. These mucosal samples were placed into 1.5 mL pre-sterilized cryovials, snap-frozen in liquid nitrogen, and stored at −80 °C. Additionally, cecal digesta was collected into 5 mL pre-sterilized cryovials, snap-frozen in liquid nitrogen, and stored at −80 °C for subsequent analysis. One cecal digesta sample from one piglet was accidentally lost during sample processing. To maintain an equal sample size across treatment groups, 5 samples from each group were randomly selected for 16S rRNA gene sequencing, resulting in 25 samples for microbiota analysis.

### 2.8. Chemical Analysis and Nutrient Digestibility

Titanium dioxide (TiO_2_) was included in the experimental diets at 0.2% as an indigestible marker for determination of apparent total tract digestibility (ATTD). Fecal samples were collected from days 19 to 21 of the experimental period. Approximately 200 g of feces was collected from each piglet each day after thorough mixing of the fecal material. At the end of the 3 d collection period, fecal samples collected from each piglet were thoroughly mixed and representative subsamples were obtained. The fecal samples were oven-dried at 65 °C for 72 h and ground to pass through a 1 mm sieve [8]. The concentrations of dry matter (DM; AOAC 934.01) [27], crude protein (CP; AOAC 990.03) [28], ether extract (EE; AOAC 920.39) [29], and crude ash (ISO 5984) [30] in both feed and fecal samples were determined. Titanium dioxide concentrations were determined according to Morgan et al. [31]. The ATTD of DM, CP, EE, and crude ash was calculated using the following equation: ATTD (%) = [1 − (TiO_2_^d^ × Nutrient^f^)/(TiO_2_^f^ × Nutrient^d^)] × 100, where Nutrient^d^ and TiO_2_^d^ are the concentrations of nutrient and TiO_2_, respectively, in the diet (g/kg DM), and Nutrient^f^ and TiO_2_^f^ are the concentrations of nutrient and TiO_2_, respectively, in the feces (g/kg DM).

### 2.9. Intestinal Histology (Hematoxylin and Eosin (H&E) Staining)

Jejunal tissues fixed in 4% paraformaldehyde underwent a series of ethanol dehydration steps before being embedded in paraffin blocks. Subsequently, the tissues were sectioned at a thickness of 8 μm and subjected to hematoxylin and eosin (H&E) staining. Microscopic images were acquired using an Echo Revolve microscope (RVL-110-G, Echo Laboratories, San Francisco, CA, USA). Villus height (VH) and crypt depth (CD) were measured from magnified images of jejunal sections. For each piglet, 3 representative sections were selected for morphological analysis, and at least 10 intact villi and crypts were randomly selected and measured across the sections. The VH-to-CD ratio (V:C) was calculated accordingly.

### 2.10. Digestive Enzyme Activities in the Jejunum

To evaluate whether ETEC K88 challenge and MP supplementation affected digestive function, the activities of lipase, trypsin, and α-amylase in the jejunal mucosa were determined using commercial assay kits (lipase, A054; trypsin, A080; and α-amylase, C016; Nanjing Jiancheng Bioengineering Institute, Nanjing, China), according to the manufacturer’s instructions.

### 2.11. Serum Analysis

To evaluate hepatic function and systemic inflammatory status, serum concentrations of alanine aminotransferase (ALT) and aspartate aminotransferase (AST) were determined using commercial kits (Nanjing Jiancheng Bioengineering Institute, Nanjing, China). Additionally, serum concentrations of TNF-α and IL-1β were quantified using commercial ELISA kits (TNF-α, CSB-E16980p; IL-1β, CSB-E06782p; Cusabio, Wuhan, China), according to the manufacturer’s instructions. The detection ranges were 0.047–3 ng/mL for TNF-α and 3.9–250 pg/mL for IL-1β.

### 2.12. Quantitative Real-Time PCR

Total RNA was extracted from the jejunal mucosa of the piglets using TRIzol reagent (Vazyme, Nanjing, China) according to the manufacturer’s instructions. The concentration and purity of the extracted RNA were quantified using a NanoDrop 2000 spectrophotometer (Thermo Fisher Scientific, Waltham, MA, USA). Subsequently, reverse transcription and qRT-PCR were performed using commercial kits (Vazyme, Nanjing, China) following the manufacturer’s protocols. The qRT-PCR assays were conducted in duplicate using a real-time PCR system (qTOWER3, Analytik Jena, Jena, Germany) to determine the mRNA expression levels of nutrient transporters and intestinal tight junction proteins. The specific primers used in this study are listed in Table 2. *β-actin* was used as the internal reference gene for normalization. Relative mRNA expression levels were calculated using the comparative 2^−ΔΔCT^ method as described by Livak and Schmittgen [32].

### 2.13. 16S rRNA Gene Amplicon Sequencing and Bioinformatics Analysis

The composition of cecal microbiota was analyzed using TGuide S96 Magnetic Soil/Stool DNA Kit (Tiangen Biotech (Beijing) Co., Beijing, Ltd.) according to the manufacturer’s instructions. Following DNA quality assessment, the V3–V4 hypervariable regions of the microbial 16S rRNA gene were amplified using universal primer pairs 338F: 5′-ACTCCTACGGGAGGCAGCA-3′ and 806R: 5′-GGACTACHVGGGTWTCTAAT-3′, as previously described [33]. High-throughput sequencing was performed on the Illumina NovaSeq 6000 platform (Illumina, San Diego, CA, USA) by Biomarker Technologies (Beijing, China). Raw reads were subjected to quality filtering using Trimmomatic (version 0.33), and chimeric sequences were identified and removed using the UCHIME algorithm (version 8.1) to obtain clean reads. These reads were subsequently clustered into operational taxonomic units (OTUs) at a 97% similarity threshold using USEARCH (version 10.0); OTUs with fewer than two total counts across all samples were excluded. Taxonomic annotation was performed using the RDP Classifier against the Greengenes database (version 13.5). Alpha diversity indices and beta diversity, visualized via principal coordinate analysis (PCoA), were calculated using QIIME2. To identify significantly enriched bacterial taxa among groups, linear discriminant analysis (LDA) effect size (LEfSe) was performed. Taxa with an LDA score > 3.0 and *p* < 0.05 were considered statistically significant.

### 2.14. Statistical Analysis

All data were analyzed using SPSS software (Version 25, SPSS Inc., Chicago, IL, USA). The individual piglet was considered the experimental unit because each piglet was housed individually. Continuous data were assessed for normality and homogeneity of variance before analysis. Data satisfying the assumptions of parametric analysis were subjected to one-way ANOVA, followed by Tukey’s multiple-comparison test. Results are expressed as the mean ± standard deviation (SD). Statistical significance was defined at *p* < 0.05, while 0.05 ≤ *p* < 0.10 was considered a trend toward significance.

## 3. Results

### 3.1. Optimal MMT to PAL Ratios for MP Synthesis

The adsorption efficiency of MMT for PAL exhibited a strong dependence on the PAL-to-MMT mass ratio. As the PAL:MMT ratio increased from 0.25:1 to 1:1 (*w*/*w*), the adsorption efficiency decreased from 63.3% to 33.4% (Figure 1B), suggesting a saturation of the cation exchange sites within the MMT at higher PAL concentrations. Conversely, the actual PAL adsorption capacity (content) within the MP increased with higher PAL loading, reaching maximum values of 245 ± 27 mg/g at a 0.75:1 ratio and 250 ± 13 mg/g at a 1:1 ratio (Figure 1C). Although the PAL content in the MP was statistically similar between the 0.75:1 and 1:1 ratios (*p* > 0.05), the adsorption efficiency was higher at 0.75:1 compared to 1:1 (43.3% vs. 33.4%, Figure 1B). Consequently, the PAL:MMT ratio of 0.75:1 was selected as the optimal synthesis ratio to maximize PAL loading while maintaining relatively high intercalation efficiency.

### 3.2. Characteristics of the MP

**Figure 2:**
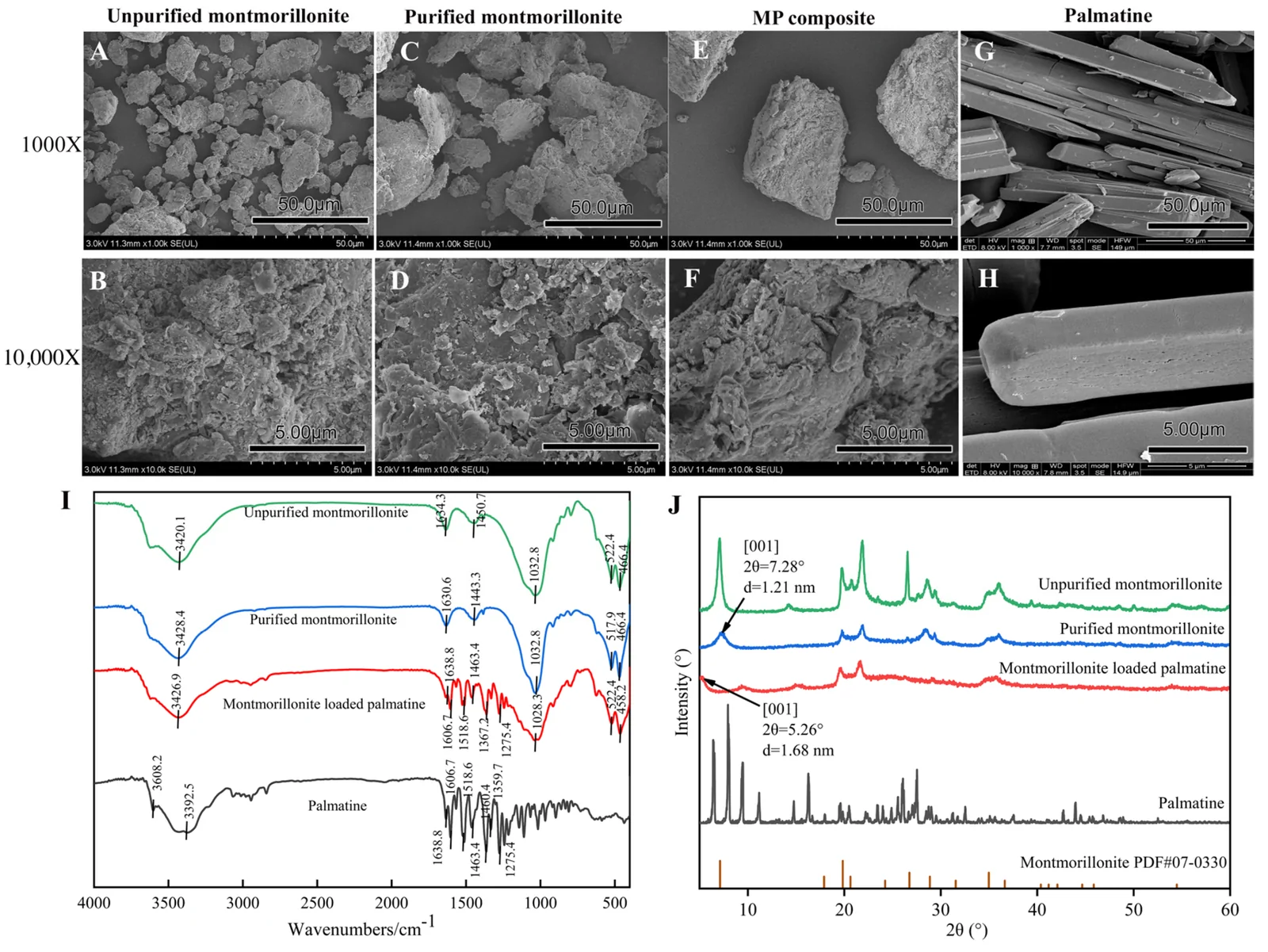
Structural characterization of the synthesized MP. (**A**–**H**) Scanning electron microscopy (SEM) images of unpurified montmorillonite, purified montmorillonite, the MP, and pure palmatine at different magnifications; (**I**,**J**) Fourier-transform infrared (FTIR) spectra and X-ray diffraction (XRD) patterns of unpurified montmorillonite, purified montmorillonite, the MP, and pure palmatine.

The morphological changes during the purification and synthesis processes were observed via SEM (Figure 2). Unpurified MMT exhibited coarse aggregates (Figure 2A). Following sedimentation purification, the MMT displayed increased morphological homogeneity, characterized by thin, flexible layers and a more crumpled texture (Figure 2C,D). Pure PAL exhibited distinctive prismatic crystalline structures, appearing as elongated rectangular cuboids with high aspect ratios, well-defined rectilinear edges, and flat facets (Figure 2G). In contrast, the MP featured thicker layers compared to the purified MMT (Figure 2E,F). Notably, the absence of PAL crystals on the surface of the MP suggests that the PAL molecules were effectively intercalated into the MMT interlayer spaces.

XRD analysis further confirmed the success of the purification and intercalation processes. After sedimentation, the diffraction peak for quartz (Figure 2J, 2θ = 26.6°) disappeared, which confirms the successful removal of quartz impurities. The basal (001) reflection of pristine MMT shifted from 2θ = 7.28° to 2θ = 5.26° after composite formation, corresponding to an increase in d_001_ spacing from 1.22 nm to 1.68 nm. This significant expansion (Δd_001_ = 0.46 nm), combined with the disappearance of PAL crystalline reflections in the composite, indicates that PAL was successfully intercalated into the interlayer galleries of the MMT, losing its bulk crystalline structure. The FTIR spectra (Figure 2I) provided additional evidence of the chemical interactions within the composite. MMT exhibited characteristic peaks at ~3620–3625 cm^−1^ (OH stretching of structural hydroxyl groups (Al-OH, Mg-OH, and Si-OH groups)), 1030–1040 cm^−1^ (Si-O-Si stretching vibration), and ~520–530 cm^−1^ and 460–470 cm^−1^, indicating Si–O–Al and Si–O–Mg bending vibrations [34]. Pure PAL showed peaks at around 1630–1600 cm^−1^ (C=C stretching in aromatic rings), 1510–1480 cm^−1^ (C=C/C=N aromatic skeletal vibrations), 1275 cm^−1^ (C–O stretching), and 1150–1050 cm^−1^ (C–N or C–O vibrations). The MP displayed characteristic PAL vibrations (e.g., aromatic C=C/C=N and C–O bands at 1518 cm^−1^ and 1275 cm^−1^) alongside shifts in the MMT bands (e.g., Si-O stretching shifting to 1028 cm^−1^), consistent with hydrogen bonding and electrostatic interactions between PA and the MMT. The simultaneous gallery expansion observed by XRD and the chemical-bonding evidence from FTIR strongly support that PAL was successfully intercalated into MMT.

### 3.3. In Vitro Release Kinetics of PAL from the MP

**Figure 3:**
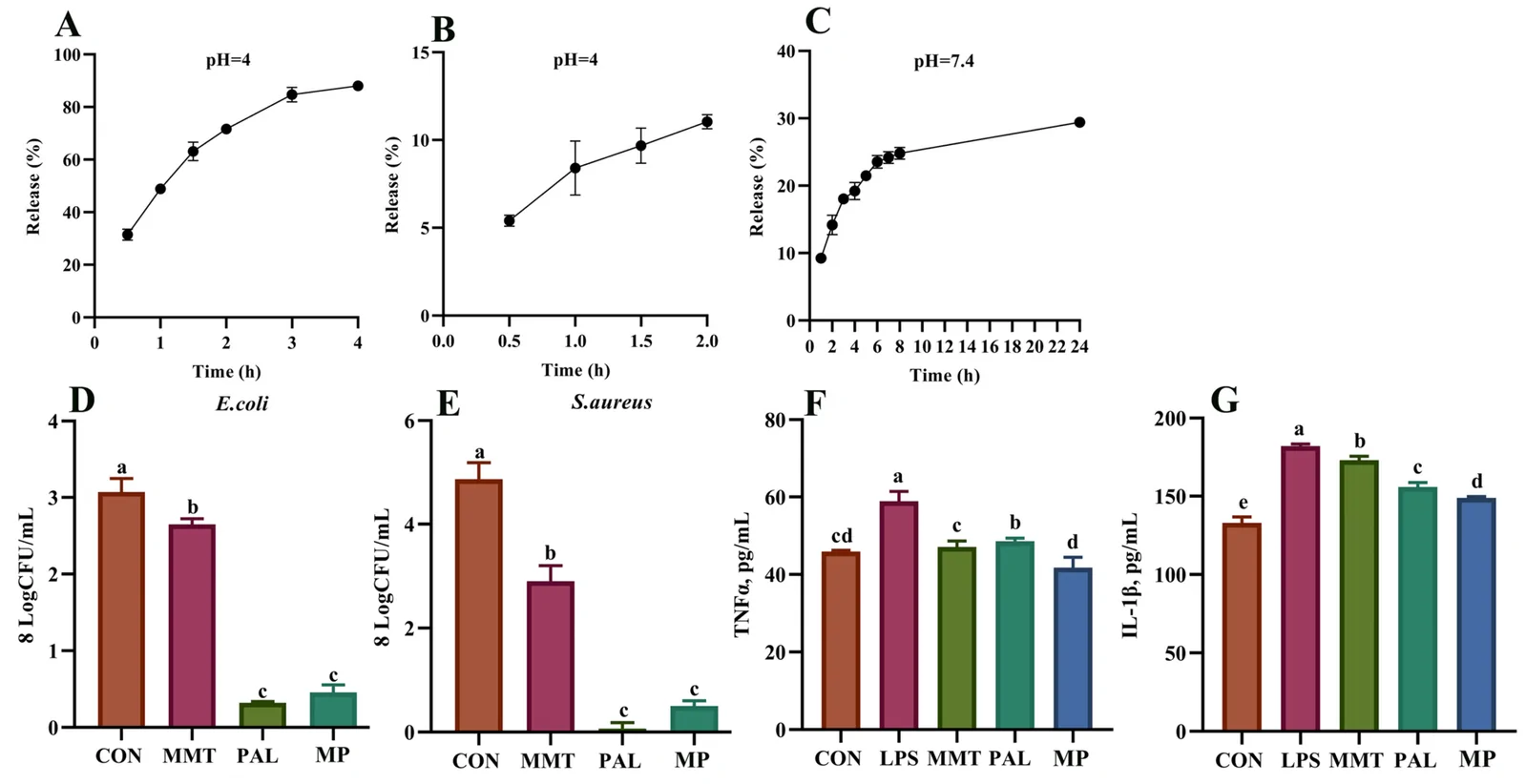
In vitro release kinetics, antibacterial efficacy, and anti-inflammatory effects of the MP. (**A**–**C**) Cumulative release rates of palmatine from the MP under simulated gastric (pH 4.0) and intestinal (pH 7.4) conditions over time; (**D**,**E**) antibacterial inhibitory effects against *E. coli* and *S. aureus* (*n* = 6); and (**F**,**G**) anti-inflammatory effects on LPS-stimulated IPEC-J2 cells, as indicated by TNF-α and IL-1β concentrations (*n* = 3). Different lowercase letters (a, b, c, d) indicate significant differences (*p* < 0.05). MMT, montmorillonite; PAL, palmatine; CON, control.

As shown in Figure 3A, pure PAL exhibited rapid release at pH 4.0, with 48.9% released within the first hour and approximately 70% released by 2 h. In contrast, the MP demonstrated significantly sustained release characteristics; at pH 4.0, only about 11% of the intercalated PAL was released within 2 h (Figure 3B). Upon transferring the MP to simulated intestinal fluid, the cumulative release reached 12% initially, gradually increasing to 23.2% at 8 h and reaching 30% by 24 h (Figure 3C).

### 3.4. Antibacterial and Anti-Inflammatory Effects of the MP

Considering that PAL was not completely released from the MP, we further evaluated the antibacterial and anti-inflammatory activities of the composite itself. As shown in Figure 3D,E, 4 mg/mL MMT, PAL, and MP significantly inhibited the growth of *E. coli*; similarly, 0.2 mg/mL of these substances significantly inhibited *S. aureus* compared to the control (*p* < 0.05). The inhibitory effects of the MP against both pathogens were comparable to those of pure PAL (*p* > 0.05), and significantly higher than those of MMT (*p* < 0.05). Furthermore, the anti-inflammatory effects of the MP were evaluated in LPS-stimulated IPEC-J2 cells. As illustrated in Figure 3F, the MP significantly reduced TNF-α concentrations induced by LPS stimulation compared to both the MMT and pure PAL treatments (*p* < 0.05). No significant difference was observed between the MP group and the control group (*p* > 0.05). Similar trends were observed for IL-1β concentrations (Figure 3G). While LPS stimulation significantly increased IL-1β levels, MMT, PAL, and MP treatments all effectively reduced these concentrations, with the MP demonstrating the most potent anti-inflammatory effect (*p* < 0.05, Figure 3G).

### 3.5. Growth Performance of Piglets Challenged with ETEC and Supplemented with the MP

The growth performance data are summarized in Table 3. Prior to the ETEC challenge (days 8–14), dietary supplementation with the MP did not influence the ADG of the piglets (*p* > 0.05). Following the ETEC challenge (days 15–21), a significant reduction in ADG was observed in the ETEC-only group compared to the control group (*p* < 0.05). However, administration of 1.5% MP mitigated the growth depression caused by the ETEC challenge, as evidenced by a significant increase in ADG and a significant reduction in the feed-to-gain (F:G) ratio during the day 15–21 period compared to the ETEC group (*p* < 0.05). No significant differences were observed in the final body weights of the piglets among all treatment groups (*p* > 0.05).

### 3.6. Nutrient Digestibility in ETEC-Challenged Piglets Supplemented with the MP

The effects of ETEC challenge and dietary MP supplementation on nutrient digestibility are presented in Table 4. Neither the ETEC challenge nor the MP supplementation significantly influenced the apparent total tract digestibility (ATTD) of dry matter or ether extract (*p* > 0.05). However, crude protein digestibility was significantly affected by the MP inclusion levels. Compared to the ETEC-only group, piglets supplemented with 1.5% MP exhibited a significant increase in the ATTD of crude protein (*p* < 0.05).

### 3.7. Intestinal Digestive Enzyme Activities and Nutrient Transporter Gene Expression

**Figure 4:**
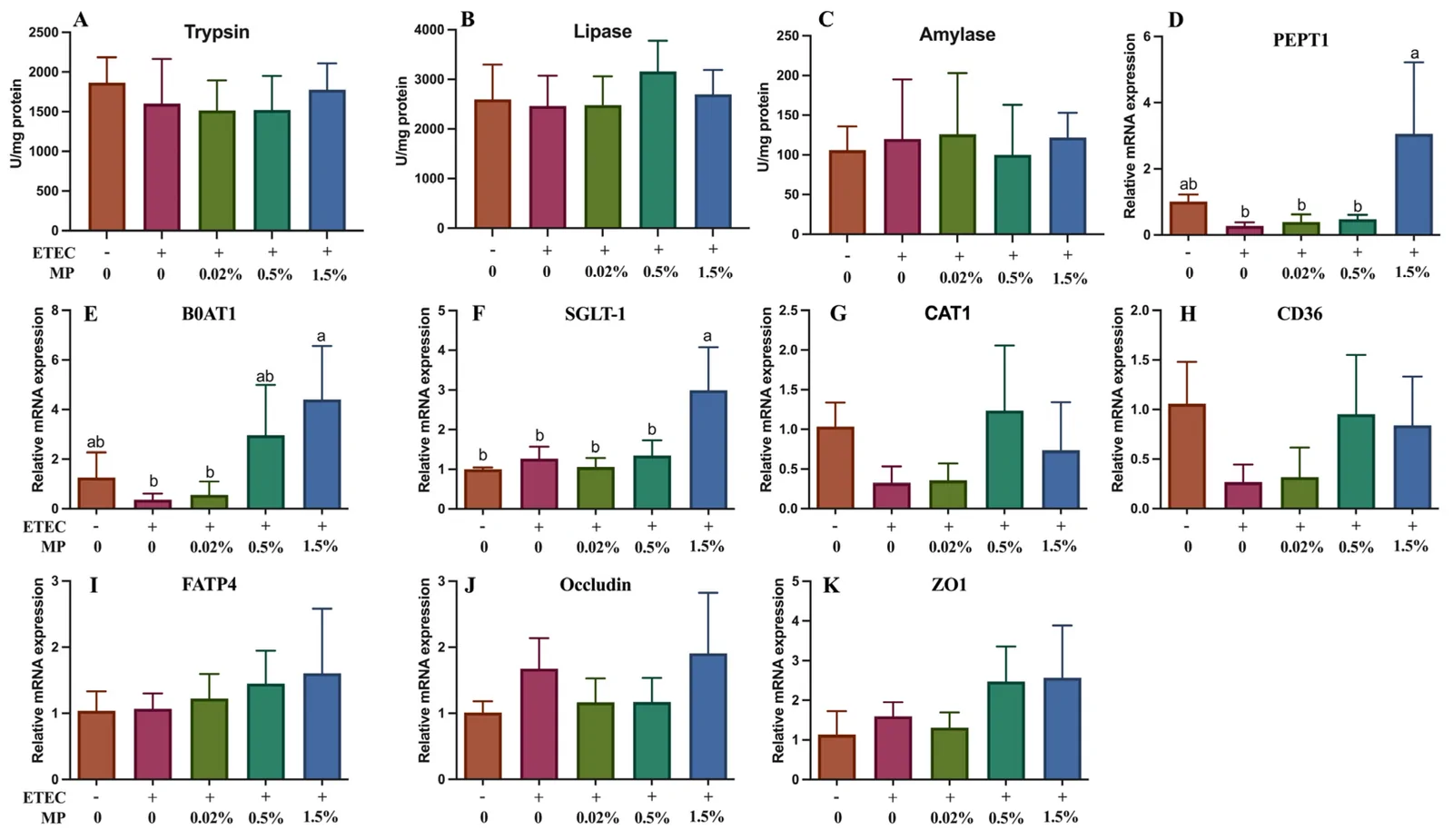
Jejunal digestive enzyme activities and nutrient transporter gene expression in ETEC-challenged piglets supplemented with the MP. (**A**–**C**) Activities of trypsin, lipase, and amylase in the jejunal mucosa. (**D**–**K**) Relative mRNA expression of *PEPT1*, *B0AT1*, *SGLT-1*, *CAT1*, *CD36*, *FATP4*, *Occludin*, and *ZO1* in the jejunum. Different lowercase letters (a, b) indicate significant differences (*p* < 0.05). *B0AT1*, sodium-dependent neutral amino acid transporter; *CAT1*, cationic amino acid transporter; *PEPT1*, peptide transporter 1; *SGLT1*, sodium-glucose cotransporter 1; *FATP4*, fatty acid transporter protein 4; *CD36*, CD36 molecule; *ZO1*, tight junction protein 1.

To investigate whether the improved crude protein digestibility was driven by enhanced enzyme secretion or upregulated nutrient transport, we first evaluated the activities of α-amylase, lipase, and trypsin in the jejunal mucosa. As shown in Figure 4A–C, no significant differences were observed among the treatment groups regarding these digestive enzyme activities (*p* > 0.05). We subsequently analyzed the mRNA expression levels of key nutrient transporters in the jejunum. As depicted in Figure 4D,E, supplementation with 1.5% MP significantly upregulated the gene expression of the peptide transporter *PEPT1* and the neutral amino acid transporter *B0AT1* in ETEC-challenged piglets compared to the ETEC-only group (*p* < 0.05); however, 0.02% MP supplementation had limited effects (*p* > 0.05). Additionally, compared to all other groups, 1.5% MP supplementation significantly increased the mRNA expression of the glucose transporter *SGLT1* (*p* < 0.05; Figure 4F). In contrast, no significant differences were observed in the mRNA expression of *CAT1*, *CD36*, *FATP4*, or the tight junction proteins *Occludin* and *ZO-1* (Figure 4G–K, *p* > 0.05).

### 3.8. Jejunal Morphology of Piglets Following ETEC Challenge and MP Supplementation

**Figure 5:**
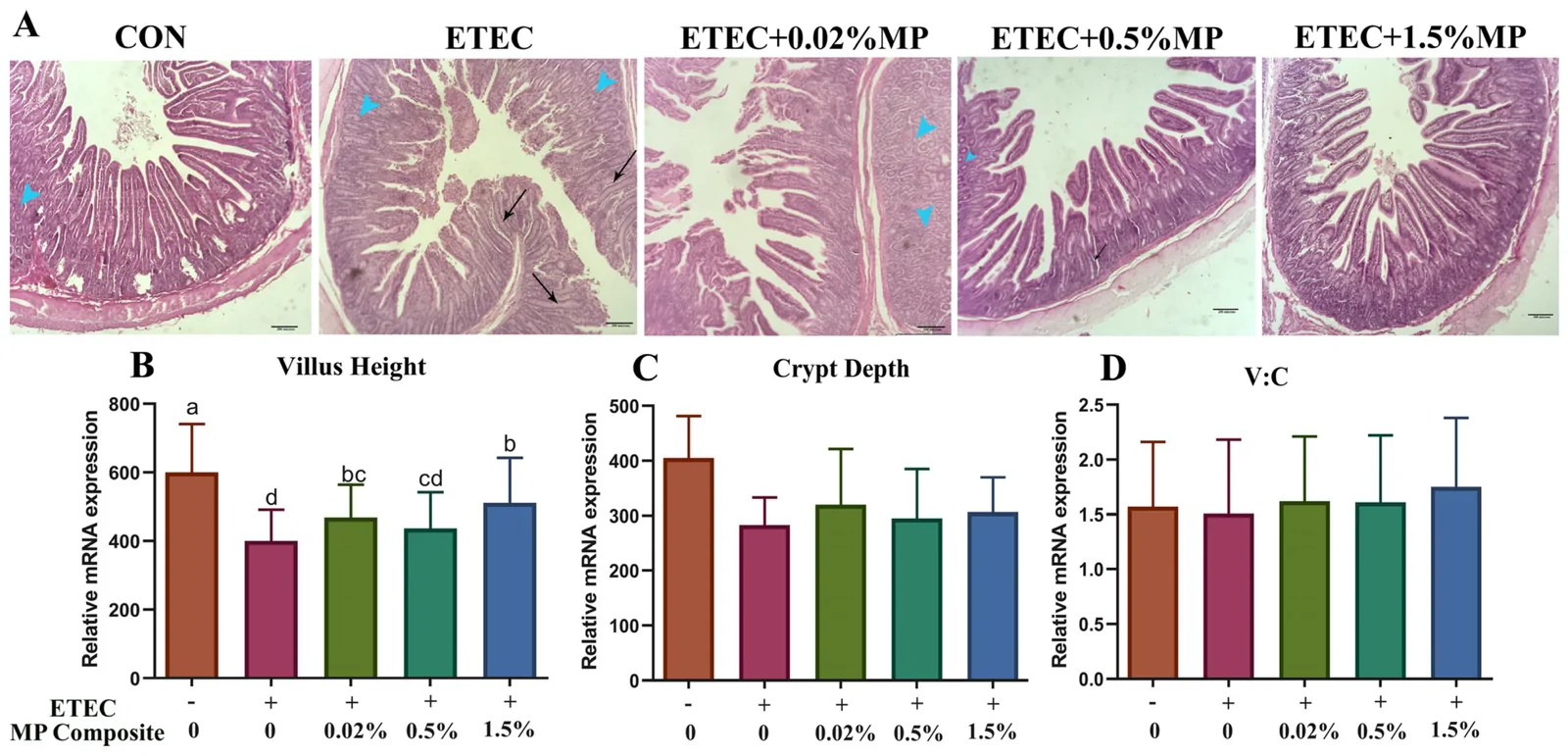
Jejunal morphology of ETEC-challenged piglets supplemented with MP. (**A**) Representative H&E-stained histological images of the jejunum across different treatment groups (scale bars = 200 μm); (**B**–**D**) morphometric analysis of villus height (VH), crypt depth (CD), and the villus-to-crypt (V:C) ratio. Arrows indicate elongated crypts, and blue arrowheads indicate a high crypt density. Different lowercase letters (a, b, c, d) indicate significant differences (*p* < 0.05).

Intestinal dysfunction remains a critical challenge in weaned piglets, as the intestine serves as the primary site for nutrient absorption and acts as a vital barrier against luminal pathogens [2]. We performed a morphometric analysis to evaluate the histological integrity of the jejunum. As illustrated in Figure 5A (blue arrowheads), a high density of crypts was observed across all treatment groups, reflecting the rapid intestinal development characteristic of piglets at this growth stage. However, piglets in the ETEC-challenged groups exhibited severe villous atrophy and elongated crypts (arrows). Compared to the control group, ETEC challenge significantly reduced jejunal VH regardless of MP supplementation (*p* < 0.05, Figure 5B). Notably, dietary supplementation with 1.5% MP significantly increased the VH of ETEC-challenged piglets compared to the ETEC-only group. There were no significant differences observed among the treatment groups regarding CD or the V:C ratio (*p* > 0.05, Figure 5C,D).

### 3.9. Serum Inflammatory Cytokines and Hepatic Enzyme Concentrations

To assess the systemic inflammatory response and hepatic function following the ETEC challenge, we measured serum cytokine and transaminase concentrations (Table 5). As shown, serum AST concentrations in the ETEC-challenged group were significantly higher than those in the control group (*p* < 0.05), indicating a degree of hepatic stress or systemic injury. However, supplementation with 1.5% MP significantly reduced serum AST concentrations compared to the ETEC-only group (*p* < 0.05).

Regarding the systemic inflammatory status, the serum concentrations of IL-1β and TNFα were significantly elevated in the ETEC-challenged group compared to the control group (*p* < 0.05). Dietary inclusion of 1.5% MP significantly lowered the serum concentrations of both IL-1β and TNFα compared to the ETEC group (*p* < 0.05).

### 3.10. Cecal Microbiota Composition and Diversity

In vitro release kinetics indicated that PAL is not fully released from the MP in the upper gastrointestinal tract, allowing a significant portion to reach the hindgut. Consequently, we analyzed the cecal microbiota to monitor the effects of ETEC challenge and MP supplementation on microbial ecology. A total of 2,000,170 pairs of raw reads were generated from 25 cecal digesta samples, yielding 1,884,118 pairs of clean reads. Each sample yielded approximately 75,365 reads on average. Based on a 97% similarity threshold, 10,717 OTUs were identified. Good’s coverage exceeded 99% for all samples, indicating sufficient sequencing depth to capture the microbial diversity present.

**Figure 6:**
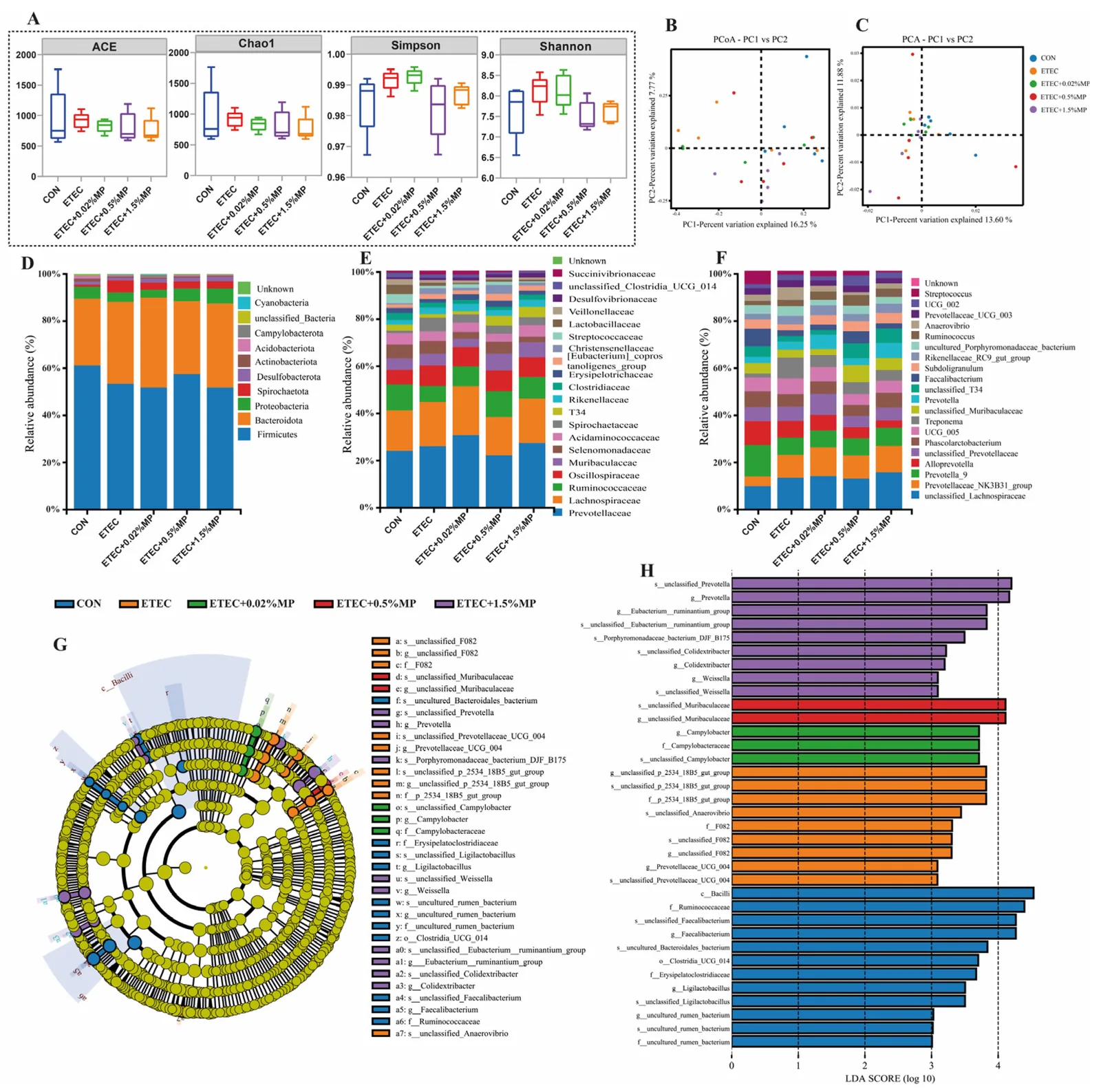
Cecal microbiota composition and diversity in ETEC-challenged piglets across different treatment groups. (**A**) Alpha diversity metrics (ACE, Chao1, Simpson, and Shannon indices). (**B**,**C**) Beta diversity evaluated via principal coordinate analysis (PCoA) and principal component analysis (PCA). (**D**–**F**) Relative abundance of the cecal microbiota at the phylum, family, and genus levels. (**G**,**H**) Linear Discriminant Analysis Effect Size (LEfSe) cladogram and LDA scores (>3.0), identifying biomarker taxa distinct to each treatment group.

Alpha diversity metrics, including ACE, Chao1, Simpson, and Shannon indices, did not differ significantly among treatment groups (*p* > 0.05; Figure 6A). However, the Simpson and Shannon indices tended to be higher in the ETEC and 0.02% MP groups compared to the other groups (*p* = 0.086 and *p* = 0.080, respectively). Principal coordinate analysis (PCoA) based on Bray–Curtis distances showed a clear separation between the control and ETEC groups (Figure 6B). ANOSIM indicated a trend toward greater inter-group than intra-group differences (*R* > 0, *p* = 0.06; Figure S2A), while PERMANOVA revealed a significant effect of treatment on microbial community structure (R^2^ = 0.2, *p* = 0.041).

The relative abundance of the cecal microbiota at phylum, family, and genus levels is presented in Figure 6D–F. The most abundant microbiota at the phylum level is Firmicutes, accounting for 54.6% of all microbiota, followed by Bacteroidota, which accounts for 33.3% of microbiota. The predominant bacterial families in the cecal microbiota were Prevotellaceae, Lachnospiraceae, Ruminococcaceae, and Oscillospiraceae, which accounted for 22.6%, 15.8%, 7.9%, and 7.0% of the total bacterial abundance, respectively. Prevotellaceae is classified within the phylum Bacteroidota, whereas Lachnospiraceae, Ruminococcaceae, and Oscillospiraceae are classified within the phylum Firmicutes. The most abundant genera in the piglets were Prevotella_9, Alloprevotella, Treponema, etc. LEfSe analysis (LDA score > 3.0) was used to identify specific microbial biomarkers (Figure 6G,H). Faecalibacterium and Ligilactobacillus were enriched in the control group, whereas Prevotellaceae_UCG_004 and unclassified_F082 were characteristic of the ETEC group. In the MP-supplemented groups, Campylobacter was enriched at the 0.02% dose, while Weissella, Colidextribacter, and Prevotella were enriched at the 1.5% dose.

## 4. Discussion

Pig production is heavily affected by piglet disease around and after weaning, and ETEC is one of the main bacterial problems because it causes post-weaning enteric disorders, poor growth, and economic loss [13]. ETEC infection not only results in ETEC colonization of the intestine, but the enterotoxins secreted by ETEC can also disrupt intestinal barrier function and induce intestinal inflammation [5]. MMT, a layered silicate, is a cost-effective feed additive frequently used in piglets for its ability to adsorb bacteria and mycotoxins and has been reported to help prevent or alleviate bacterial-associated diarrhea, particularly that caused by ETEC [35]. MMT is widely used as a carrier in antibacterial materials because its layered structure allows adsorption, intercalation, and controlled release of bioactive molecules. At the same time, MMT can also be used as a carrier loaded with other functional substances to prolong their efficacy [36]. This controlled release extends the duration of their functional effects. However, due to its layered characteristics, when blended with feedstuff, it may adsorb other micro-nutrients in the diet, such as minerals. Consequently, higher doses (more than 1%) might induce adverse effects on the health of the pigs, leading to impaired liver health, poor production performance, and lower bone mineral accumulation [18,37]. Combining MMT with other functional substances could prevent the adsorption of micro-nutrients in the feed. Palmatine, an alkaloid traditionally extracted from medical plants such as Coptis chinensis Franch and Daemonorops margaritae (Hance) Becc., has strong antibacterial [21], anti-inflammatory, and antioxidant effects [38]. However, PAL shows limited oral bioavailability because intestinal P-glycoprotein-mediated efflux and first-pass metabolism restrict its absorption [23]. Because PAL may exert biological effects at the intestinal epithelial interface, sustained delivery of PAL by a carrier such as MMT may help maintain its availability in the intestine and prolong its local effects. Given that ETEC challenge in piglets involves severe intestinal inflammation, we therefore synthesized a PAL-MMT composite. This novel feed additive was designed to synergistically adsorb bacteria and toxins, exert bactericidal activity, and provide anti-inflammatory benefits. 

Because MMT exhibits excellent physical and chemical swelling properties in water—resulting in increased volume and basal spacing [39]—a hydration dispersion method was employed in this study to intercalate PAL into the MMT. This process generates no secondary pollution, making it environmentally friendly and easily scalable. FTIR and XRD results confirmed that PAL was successfully intercalated into the layers of MMT, resulting in enlarged interlayer spacing and increased particle size in the MP. In vitro release kinetics demonstrated that around 23% of the PAL was released within 8 h at 37 °C and pH 7.4. This release rate is consistent with other drug-MMT composites [40]. The negative surface charge of MMT interacts with cationic PAL molecules, forming hydrogen bonds and electrostatic interactions that create a strong binding affinity, thereby facilitating the sustained release of PAL from the MP. Although 30% of the PAL was released after 24 h, 70% remained stably retained within the composite. At equivalent concentrations, the MP exhibited superior antibacterial efficacy compared to MMT alone and performed similarly to pure PAL. Previous studies have reported that modified MMT demonstrates enhanced adsorption and antibacterial properties [41]. Because the MMT in the composite was purified and modified with PAL to expand its interlayer spacing, its surface and internal adsorption capacities were likely enhanced. Furthermore, electrostatic interactions with bacterial membranes may facilitate closer contact and potentiate the inhibitory effects of PAL. Moreover, the MP showed stronger anti-inflammatory effects than either MMT or PAL alone in LPS-challenged IPEC-J2 cells, suggesting that the intact composite retains significant anti-inflammatory and antibacterial activities even before full PAL release in the intestine. It has been reported that modified MMT can downregulate Toll-like receptors (TLRs), thereby reducing the expression of pro-inflammatory cytokines such as TNF-α and IL-1β [42]. Therefore, the anti-inflammatory effects of MP in LPS-stimulated IPEC-J2 cells may be associated with modulation of TLR-related inflammatory responses.

Despite containing a lower absolute amount of PAL, the MP exerts antibacterial and anti-inflammatory effects similar to those of pure PAL at equivalent total concentrations. Previous research indicated that an oral dose of 10 mg/kg of PAL reduced *E. coli* colonization in the organs of infected mice [21]. Because the standard inclusion rate of PAL extracts in animal production is around 200 mg/kg, we evaluated an initial MP dose of 0.02% to determine if efficacy could be maintained at a lower threshold. Furthermore, supplementing weaned piglet diets with 0.2% MMT has been shown to promote intestinal development and health [43]. Conversely, MMT inclusion rates exceeding 2% can induce adverse physiological effects in piglets [37]. Therefore, dietary inclusion levels of 0.5% and 1.5% MP were selected for the ETEC-challenged piglets in this study.

ETEC-induced intestinal dysfunction is a major driver of impaired growth performance in post-weaning piglets, primarily through disruption of epithelial barrier integrity, induction of inflammation, and alteration of microbial homeostasis [5,9]. In the present study, ETEC K88 challenge significantly reduced growth performance, as evidenced by decreased average daily gain and increased feed-to-gain ratio, which is consistent with previous reports. This performance decline is largely attributable to impaired nutrient utilization, as intestinal inflammation and mucosal damage compromise digestive and absorptive functions [44]. Accordingly, we observed a reduction in crude protein digestibility in ETEC-challenged piglets, providing a direct explanation for the observed growth depression. The jejunum, as the primary intestinal segment for nutrient digestion and absorption in piglets, is particularly susceptible to ETEC-induced injury [7]. In line with this, histological analysis revealed reduced villus height in the jejunum following ETEC challenge, indicating diminished absorptive surface area. Notably, dietary supplementation with the MP significantly improved crude protein digestibility in a dose-dependent manner. The improvement was not associated with changes in digestive enzyme activity, but rather with a marked upregulation of nutrient transporter expression, particularly *PEPT1* and *B0AT1*. These findings suggest that the MP improves protein utilization in ETEC-challenged piglets, potentially by enhancing intestinal absorptive capacity rather than promoting enzymatic digestion. The upregulation of these nutrient transporters likely stems from the overall protective effect on intestinal health afforded by the MP. MMT has been widely reported to enhance gut barrier function in piglets [43], while PAL exerts protective effects on intestinal epithelial structure and promotes mucosal recovery [22]. Their combination in the MP may therefore create a more favorable intestinal environment that supports transporter expression and function. Similar regulatory effects have been reported for modified MMT, such as copper-loaded MMT, which enhances *PEPT1* and *B0AT1* expression in broilers [19]. Collectively, these results indicate that the MP improves nutrient utilization in ETEC-challenged piglets primarily through preservation of intestinal structure and enhancement of transporter-mediated absorption.

Beyond direct colonization and barrier impairment, ETEC-induced systemic inflammation is a critical factor driving morbidity and reduced performance in swine [5]. In this study, the elevation of serum pro-inflammatory cytokines following ETEC challenge confirmed the induction of systemic inflammation. Dietary inclusion of the MP significantly attenuated these serum cytokine levels, an effect consistent with its observed anti-inflammatory activity in LPS-treated intestinal epithelial cells. Pure PAL is known to possess strong anti-inflammatory and mucosal-protective properties [38]. Similarly, berberine—a structural analog of PAL—alleviates ETEC-induced intestinal inflammation in weaned piglets [10]. The reduction in serum inflammatory cytokines observed with up to 1.5% MP supplementation strongly aligns with our in vitro findings. Concurrently, MMT has the capacity to adsorb luminal bacteria and reinforce mucosal integrity, thereby minimizing systemic LPS exposure and subsequently lowering pro-inflammatory cytokine production [35]. At the same time, these anti-inflammatory benefits are partially mediated by the progressive release of PAL and MMT; the in vitro data suggest that the intact, unreleased composite also actively suppresses inflammation. In agreement with our findings, andrographolide-loaded MMT has been shown to exert potent anti-inflammatory effects in ETEC-challenged mice [15]. Ultimately, the mitigation of inflammation helps preserve intestinal integrity, creating a physiological environment conducive to optimal nutrient absorption.

ETEC challenge disrupts the homeostasis of the piglet intestinal microbiota, typically characterized by reduced alpha diversity and a destabilized community structure [12,45]. Accordingly, we utilized 16S rRNA sequencing to evaluate the cecal microbiota of ETEC-challenged and MP-treated piglets. In the present study, the α diversity of cecal microbiota was not significantly changed by ETEC or MP supplementation. However, PCoA analysis showed that the cecal microbial communities in piglets receiving the higher dose of MP separated distinctly from both the control and ETEC-challenged groups. The relative abundance of Faecalibacterium, a genus known to include fiber-degrading and butyrate-producing bacteria [11], was higher in the control group. Previous studies have reported that greater Faecalibacterium abundance is associated with higher gut microbial diversity and improved production performance in weaned piglets [46]. Weissella, a genus comprising several lactic acid bacteria with potential probiotic properties, has also been associated with gut health. For example, *Weissella cibaria* has been reported to attenuate intestinal inflammation [47]. Notably, LEfSe analysis identified Weissella as a discriminative microbial feature of the 1.5% MP group. The observed difference in Weissella abundance may be relevant to the intestinal and inflammatory responses observed in the 1.5% MP group, although a direct relationship cannot be established from the present data. Similarly, Colidextribacter, which has been reported to exhibit potential prebiotic-like effects [3], showed a difference in relative abundance following 1.5% MP supplementation. Collectively, the ETEC challenge was associated with alterations in cecal microbial community composition, whereas higher levels of dietary MP supplementation were associated with shifts in specific bacterial genera that may be relevant to intestinal health. These findings suggest that MP may modulate ETEC-associated microbial alterations, although the functional significance of these changes and their relationship with intestinal and systemic responses require further investigation.

Notably, the relatively small sample size (*n* = 6 per treatment) may have limited the statistical power to detect treatment effects, particularly for outcomes with substantial biological variability, such as gut microbiota composition. Therefore, nonsignificant findings should not necessarily be interpreted as evidence of the absence of an effect. Larger-scale studies will be required to validate the present findings and provide more robust estimates of treatment effects.

## 5. Conclusions

In summary, the successful intercalation of palmatine into montmorillonite generated a novel MP characterized by sustained-release properties and potent in vitro antibacterial and anti-inflammatory activities. In vivo, dietary supplementation with the MP—particularly at an inclusion dose of 1.5%—effectively mitigated the adverse physiological effects of ETEC K88 infection in weaned piglets. The composite improved intestinal health by alleviating ETEC-induced villus atrophy and upregulating the mucosal expression of key nutrient transporters. The observed mucosal protection was associated with improved apparent crude protein digestibility and enhanced overall growth performance in ETEC K88-challenged piglets. Furthermore, the MP was associated with reduced serum AST activity, attenuated pro-inflammatory cytokines (TNF-α and IL-1β), and modulation of the cecal microbial community. Collectively, these findings indicate that MP has potential as a nutritional strategy for supporting intestinal health and attenuating inflammatory responses in ETEC K88-challenged piglets, although further studies are warranted to establish its in vivo mechanisms of action and efficacy under post-weaning conditions.

## Figures and Tables

**Table 1:** Composition and nutrient levels of the basal diet (as-fed basis).

Ingredient	Content (%)	Nutrient Levels ^#^	Content
Corn	63.7	DE, Mcal/kg	3.49
Soybean meal	14.0	ME, Mcal/kg	3.18
Extruded soybean	14.0	Crude protein, %	18.7
Fish meal	2.0	Ca, %	0.72
L-Lys HCl	0.6	P, %	0.61
Whey powder	2.0	Lysine, %	1.43
Limestone	0.7	Methionine, %	0.32
CaHPO4	0.9		
NaCl	0.4		
Soybean oil	1.6		
Vitamin Premix ^1^	0.02		
Mineral Premix ^2^	0.08		
Total	100		

^1^ Premix supplied per kilogram of complete diet: Vitamin A, 6450 IU; Vitamin D3, 1520 IU; Vitamin E, 39.53 IU; Vitamin K3, 2 mg; Vitamin B1, 1.61 mg; Vitamin B2, 5 mg; Vitamin B6, 2.55 mg; Vitamin B12, 20 μg; D-biotin, 120 μg; Niacin, 19.6 mg; D-pantothenic acid, 12.06 mg; Folic acid, 0.99 mg; and Ethoxyquin, 0.1 mg. ^2^ Premix supplied per kilogram of complete diet: Cu, 20 mg; Fe, 104 mg; Mn, 12 mg; Zn 64 mg; I, 0.8 mg; and Se, 0.4 mg. ^#^ Calculated.

**Table 2:** Primer sequences of housekeeping and target genes.

Gene Name	Primer Sequences, 5′ to 3′	Product Size
*B0AT1*	F: ACAACAACTGCGAGAAGGAC R: GATAAGCGTCAGGATGTTCG	149
*CAT1*	F:TGCCCATACTTCCCGTCC R:GGTCCAGGTTACCGTCAG	192
*PEPT1*	F:GGATAGCCCTGTACCCCAAGCT R:CATCCTCCACGTGCTTCTTGA	74
*SGLT1*	F:AAAAATTGCCTGCACCGTCC R:CGCAATCCATTGGGCATGAG	114
*FATP4*	F:CCTCGCAGACGGAACTGAAC R:GTCTGGACCATTTCATCCCCG	121
*CD36*	F:GGAGAAAAGATCACTACCATCATGAG R:CTCCTGAAGTGCAATGTACTGACA	78
*ZO1*	F: CCAGGGAGAGAAGTGCCAGTAGG R: TTTGGTGGGTTTGGTGGGTTGAC	105
*Occludin*	F: GCACCCAGCAACGACAT R: CATAGACAGAATCCGAATCAC	102
*β-actin*	F: CGCAAGTACTCCGTGTGGAT R: GTCGTACTCCTGCTTGCTGA	87

F, forward; R, reverse; *B0AT1*, sodium-dependent neutral amino acid transporter; *CAT1*, cationic amino acid transporter; *PEPT1*, peptide transporter 1; *SGLT1*, sodium-glucose cotransporter 1; *FATP4*, fatty acid transporter protein 4; *CD36*, CD36 molecule; *ZO1*, tight junction protein 1.

**Table 3:** Growth performance of piglets treated with ETEC and MP.

Items	Treatment					*p*-Value
	CON	ETEC	0.02%MP	0.5%MP	1.5%MP	
Initial BW, kg	14.9 ± 4.04	15.1 ± 3.37	15.4 ± 2.74	15.2 ± 2.46	15.0 ± 2.15	0.99
Final BW, kg	27.9 ± 4.43	25.6 ± 3.99	26.3 ± 5.17	26.8 ± 5.11	29.7 ± 3.56	0.64
ADG, g/d						
0–7 d	431 ± 94.9	393 ± 197.5	346 ± 226.2	442 ± 177.2	570 ± 115.0	0.25
8–14 d	771 ± 121.3	678 ± 105.3	733 ± 154.1	669 ± 112.8	876 ± 82.8	0.07
15–21 d	695 ± 81.0 ^a^	462 ± 96.2 ^b^	529 ± 96.8 ^ab^	575 ± 182.8 ^ab^	697 ± 129.9 ^a^	0.02
ADFI, g/d						
0–7 d	920 ± 130.7 ^ab^	735 ± 73.9 ^ab^	655 ± 254.1 ^b^	776 ± 254.0 ^ab^	1049 ± 163.0 ^a^	0.03
8–14 d	1455 ± 271.5	1287 ± 177.8	1190 ± 376.4	1110 ± 225.6	1608 ± 229.5	0.18
15–21 d	1125.5 ± 95.0	943.1 ± 184.9	852.7 ± 200.8	869.9 ± 264.9	985 ± 135.0	0.07
F:G						
0–7 d	2.18 ± 0.32	2.00 ± 0.52	2.36 ± 1.30	1.80 ± 0.18	1.88 ± 0.36	0.68
8–14 d	1.89 ± 0.15	1.91 ± 0.20	1.59 ± 0.27	1.66 ± 0.15	1.83 ± 0.26	0.10
15–21 d	1.64 ± 0.22 ^ab^	2.05 ± 0.11 ^a^	1.60 ± 0.10 ^ab^	1.52 ± 0.12 ^ab^	1.43 ± 0.12 ^b^	0.03

Data expressed as mean ± standard deviation (*n* = 6). ^a^, ^b^ within a row; means with different lowercase superscript letters differ significantly (*p* < 0.05). CON, control group; ETEC, *E. coli*-challenged group; MP, montmorillonite-loaded palmatine composite treated in ETEC-challenged groups; BW, body weight; ADG, average daily weight gain; ADFI. average daily feed intake; F:G. feed-to-weight gain conversion ratio.

**Table 4:** Nutrient digestibility in ETEC-challenged piglets supplemented with MP.

Items	Dry Matter (%)	Crude Ash (%)	Ether Extract (%)	Crude Protein (%)
Treatment				
CON	81.4 ± 3.57	64.1 ± 7.78	69.8 ± 6.53	83.0 ± 2.00 ^ab^
ETEC	81.8 ± 1.99	60.9 ± 7.78	69.2 ± 2.98	79.2 ± 1.52 ^b^
0.02%MP	81.1 ± 3.22	62.6 ± 7.19	72.9 ± 6.87	80.4 ± 3.54 ^ab^
0.5%MP	80.5 ± 4.63	55.0 ± 11.55	73.2 ± 7.29	81.3 ± 2.91 ^ab^
1.5%MP	83.0 ± 2.46	53.6 ± 8.15	74.8 ± 5.48	84.5 ± 2.22 ^a^
*p* value	0.81	0.25	0.54	0.03

Data expressed as mean ± standard deviation (*n* = 6). ^a^, ^b^ Values within a column with different superscripts differ significantly at *p* < 0.05. CON, control group; ETEC, *E. coli*-challenged group; MP, montmorillonite-loaded palmatine composite treated in ETEC-challenged groups.

**Table 5:** Effects of the MP on serum pro-inflammatory cytokines and hepatic enzyme concentrations in ETEC K88-challenged piglets.

Items	ALT (U/L)	AST (U/L)	IL-1β (pg/mL)	TNFα (pg/mL)
Treatment				
CON	68.6 ± 5.17	54.5 ± 9.93 ^bc^	177.7 ± 23.70 ^b^	45.5 ± 3.08 ^c^
ETEC	65.5 ± 15.20	89.2 ± 11.56 ^a^	223.4 ± 9.18 ^a^	57.6 ± 5.57 ^a^
0.02%MP	70.1 ± 13.14	81.1 ± 12.36 ^a^	219.3 ± 7.79 ^a^	52.5 ± 2.41 ^ab^
0.5%MP	61.7 ± 5.66	72.8 ± 12.37 ^ab^	189.7 ± 16.22 ^b^	52.6 ± 2.06 ^ab^
1.5%MP	56.2 ± 12.11	46.8 ± 16.80 ^c^	174.3 ± 15.91 ^b^	47.4 ± 2.62 ^bc^
*p*-Value	0.31	0.01	0.01	0.01

Data expressed as mean ± standard deviation (n = 6). ^a^, ^b^, ^c^ Values within a column with different superscripts differ significantly at *p* < 0.05. CON, control group; ETEC, *E. coli*-challenged group; MP, montmorillonite-loaded palmatine composite treated in ETEC-challenged groups. ALT (GPT), glutamic pyruvic transaminase; AST (GOT), glutamic oxaloacetic transaminase.

## Data Availability

The data presented in this study are available on request from the corresponding author.

## Supplementary Materials

The following supporting information can be downloaded at https://www.mdpi.com/article/10.3390/ani16182868/s1. Figure S1: Effects of different concentrations of (A) palmatine (PAL), (B) montmorillonite (MMT), and (C) the montmorillonite-palmatine composite (MP) on the viability of IPEC-J2 cells. Cell viability was determined following treatment with increasing concentrations (0–100 μg/mL) of each compound; Figure S2: ANOSIM (A) and PERMANOVA (B) analysis of cecal microbiota in different treatment groups.

## Abbreviations

The following abbreviations are used in this manuscript:

B0AT1	Sodium-dependent neutral amino acid transporter
CAT1	Cationic amino acid transporter
CD36	CD36 molecule
ETEC	Enterotoxigenic *Escherichia coli*
FATP4	Fatty acid transporter protein 4
MMT	Montmorillonite
MP	palmatine-intercalated montmorillonite
PAL	Palmatine
PEPT1	Peptide transporter 1
SGLT1	Sodium-glucose cotransporter 1
ZO1	Tight junction protein 1
