# Supplementary material for: Palmatine-Intercalated Montmorillonite Improves Intestinal Health and Attenuates Systemic Inflammation in ETEC-Challenged Piglets
Source: Animals (Basel). 2026 Sep 11;16(18):2868. doi: 10.3390/ani16182868 (PMC13603380; doi:10.3390/ani16182868)
Supplement: Supplementary file 1 [file animals-16-02868-s001.zip › animals-4512709-supplementary.pdf]

Supplementary Materials

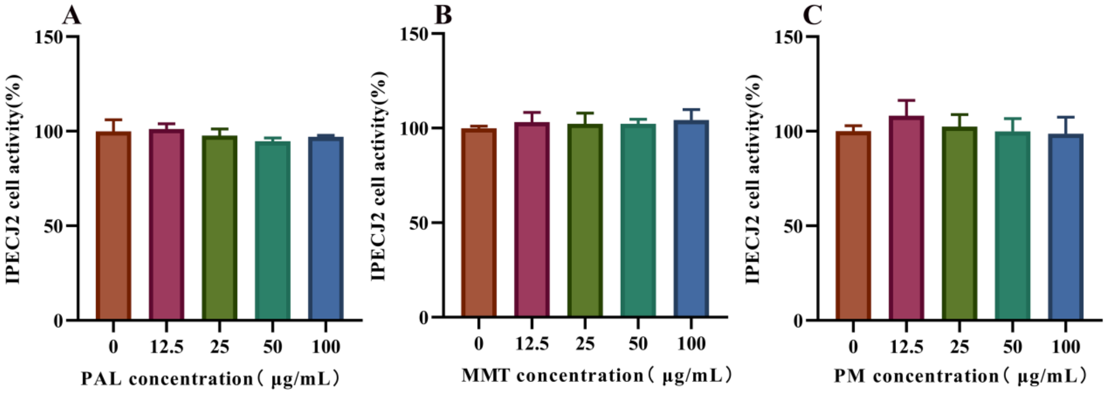

Figure S1. Effects of different concentrations of (A) palmatine (PAL), (B) montmorillonite (MMT), and (C) the montmorillonite-palmatine composite (MP) on the viability of IPEC-J2 cells. Cell viability was determined following treatment with increasing concentrations (0-100 μg/mL) of each compound.

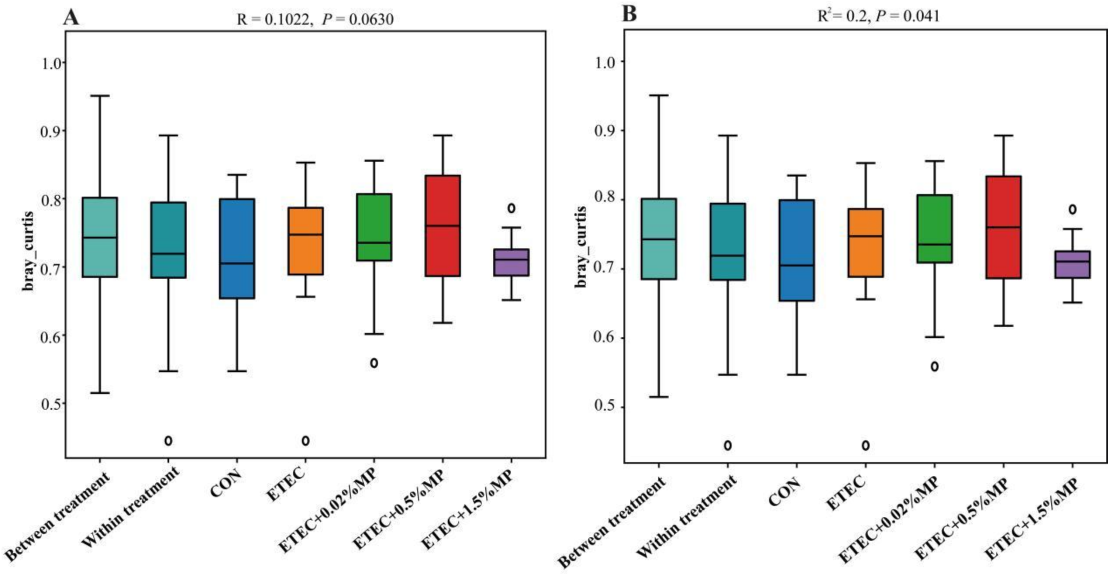

Figure S2. ANOSIM (A) and PERMANOVA (B) analysis of cecal microbiota in different treatment groups.
